# Leadership as an Emergent Feature in Social Organizations: Insights from A Laboratory Simulation Experiment

**DOI:** 10.1371/journal.pone.0166697

**Published:** 2016-12-14

**Authors:** Luis Curral, Pedro Marques-Quinteiro, Catarina Gomes, Pedro G. Lind

**Affiliations:** 1 Centro de Investigação em Ciência Psicológica, Faculdade de Psicologia, Universidade de Lisboa, Lisboa, Portugal; 2 William James Center for Research, ISPA-IU, Lisboa, Portugal; 3 Centro de Administração e Políticas Públicas, Instituto Superior de Ciências Sociais e Políticas, Universidade de Lisboa, Lisboa, Portugal; 4 Institut für Physik, Universität Osnabrück, Osnabrück, Germany; Universidad Rey Juan Carlos, SPAIN

## Abstract

Recent theoretical contributions have suggested a theory of leadership that is grounded in complexity theory, hence regarding leadership as a complex process (i.e., nonlinear; emergent). This article tests if complexity leadership theory promotes efficiency in work groups. 40 groups of five participants each had to complete four decision making tasks using the city simulation game SimCity4. Before engaging in the four decision making tasks, participants received information regarding what sort of leadership behaviors were more adequate to help them perform better. Results suggest that if complexity leadership theory is applied, groups can achieve higher efficiency over time, when compared with other groups where complexity leadership is not applied. This study goes beyond traditional views of leadership as a centralized form of control, and presents new evidence suggesting that leadership is a collective and emergent phenomenon, anchored in simple rules of behavior.

## Introduction

In the few hours that followed hurricane Katrina in 2005, groups of self-organized citizens coordinated themselves to rescue the victims and take them to dry land, while others built improvised facilities (e.g., hospitals) to accommodate the injured and homeless [1]. In contrast, in the week that followed this event formal action and command protocols failed to deliver a timely solution to the calamity. The complexity of the scenario after the Katrina was so high that centralized forms of leadership were insufficient to deliver an efficient response [2]. Whereas centralized leadership structures proved unable to provide immediate solutions, decentralized forms of leadership led to the emergence of one self-organized complex adaptive system that was more efficient coping with the situation [3].

Traditionally, leadership is understood as a direct influencing process where leaders are the motivating entity that moves or directs followers to action, ultimately ending in the achievement of goals. Leadership is regarded as a centralized form of control where one individual exerts power and influence upon others [4]. However, events such as hurricane Katrina have challenged traditional perspectives of leadership as they provide anecdotal evidence for the possibility that the most efficient leadership system is one where leadership is a decentralized and self-organized phenomenon as opposed to an individual centralized form of control. Therefore, we ask: Can decentralized and self-organized forms of control be more efficient than centralized forms of organizing and controlling? Answering this question is important to inform scholars and practitioners about alternative leadership approaches, one that fits best in today’s complex, fast changing, and often unpredictable world.

In this paper our goal is to examine to what extent practicing leadership as a decentralized and self-organized form of organization leads to higher efficiency [5]. Similarly to the Katrina event, in contemporary organizations unpredictable situations challenge leaders’ capacity to cope with a performance landscape that is continuously changing [6]. This suggests that new ways to theorize leadership might be more adequate. One possible way is by framing leadership within complexity theory [7].

### Theoretical Background

Research on the nature of leadership has been particularly fruitful within the literature of evolutionary games [8, 9, 10]. In evolutionary games, the behaviors (e.g. leadership, coordination) in which participants engage change and are influenced by the game environment [11]. For instance, Guastello [9] examined how leadership emerges in coordination-intensive groups performing evolutionary game tasks such as Stag Hunt, thus finding support for the hypothesis that leadership is a complex process where emergence can be described by means of a swallowtail catastrophe mathematical function. Furthermore, Boos et al. [8] used the HoneyComb game-simulation paradigm to test how leadership and coordination emerge in human groups where participants can only rely on their visual perception to coordinate with others. The authors found that the rules of swarming behavior observed in other species like birds or bees also apply to humans (i.e., humans coordinate their movement, hence synchronizing it, by observing others and adjusting their own moving patterns), and that leadership emergence obeys to simple rules based on the visual perception of the local movement.

In line with the above mentioned, the theory of complex systems regards the study of non-trivial emergent behavior, i.e. a global behavior observed in a system which cannot be derived from its elementary rules of constitutive parts [12]. Although complexity theory is an established field in the mathematical and live sciences, the use of complexity theory as a theoretical and methodological framework to understand psychological phenomena such as leadership is relatively new [9].

In short, in the present study, we will use evolutionary games as a general paradigm to understand leadership as a complex process by means of tasking participants with a collaborative game (i.e., SimCity4) in which the best outcome can only be achieved if participants are able to coordinate their actions during the game [10,11]. Additionally, complexity leadership theory will be regarded as the theoretical framework within which we will develop and test the premise that groups are more efficient when leadership is an emergent feature of social organizations [5].

Specifically, complexity leadership theory, based on complexity theory, refers to the study of the interactive dynamics of complex adaptive systems (CAS) that are embedded in a context of traditional large organization systems [5]. Complex adaptive systems are the basic unit of analysis in complexity theory, and represent complex networks of individuals that interact and are interdependent towards a common goal (e.g. organizations; teams). Complexity leadership theory does not discard the existence of individual formal leaders [7], but it emphasizes a perspective where leadership is seen as an observable feature that emerges from simple rules of interaction between individuals [13]. Complexity leadership theory suggests that randomness prevents individual leaders from predicting, and closely controlling the future. Randomness creates an environment where the impact of the actions of single isolated agents on organizational efficiency is trivial. In order to gain efficiency, rather than centralizing power and decision making on a minority of individuals, leadership should look for diminishing control upon others and encouraging individuals to openly communicate and share information across the entire organizational structure [14].

### Research Hypothesis

Complexity leadership influences organizational efficiency due to the existence of individuals with at least one of three fundamental functions: administrative function, adaptive function, and enabling function. Individuals with an administrative function focus on alignment and control. The administrative function refers to the hierarchical/bureaucratic managerial roles of the organization, which include managerial tasks such as planning and coordinating activities to accomplish organizational appointed outcomes, in an efficient and effective manner [7]. Additionally, individuals with an adaptive function foster change and adaptability. They do not act in an isolated manner, but rather they act as an informal collective that exchanges information to promote change [15]. To generate change in the system, individuals with adaptive functions engage in actions that are adaptive, creative, and learning oriented. However, in order to evolve and have an impact in organizational efficiency these actions need to be recognized by others within the organization as being relevant for the organizational system [7]. Finally, individuals with an enabling function help to incorporate adaptive outcomes into the formal structure of the organization. Enabling leadership catalyzes organizational efficiency by fostering enabling conditions that help manage the entanglement between the administrative and adaptive leadership functions of the system. By entanglement we mean the dynamic relationship between the formal top-down administrative forces such as bureaucracy, and the informal bottom-up complex adaptive forces of the adaptive agents of the system. Enabling leadership acts as a mediating function as it decides which outputs should flow across organizational levels and be exchanged between individuals with adaptive and administrative functions [5]. In doing this, enabling leadership creates a healthy and secure environment where the adaptive function can strive for the goals and mission of the organization, which are managed by the administrative function.

The administrative, adaptive and enabling leadership functions are intertwined in a way that promotes the emergence of CAS and facilitates efficiency. Beyond anecdotal cases such as Katrina’s in 2005, or the survival of the 33 Chilean miners buried in San José’s Mine in 2010, few studies have provided empirical support to the theoretical argument saying that leadership should rather be a collective process where leadership behaviors emerge and are shared in the team (e.g. [5, 16, 17]). In one study with self-managed virtual teams Carte, Chidambaram and Becker [18] examined how the emergence of leadership behaviors in self-managed virtual teams contributed to overall performance. They found that the best performing teams were those where emergent leadership behaviors specifically addressed more relevant team task needs. In another study with healthcare teams Fitzgerald, Ferlie, McGiven, and Buchanan [19] tested if adopting a leadership system that promoted self-organized behavior led to higher efficiency. Fitzgerald and colleagues found that when change-oriented leadership roles were encouraged (which, under complexity leadership theory, means that there is an entanglement between the administrative and adaptive leadership functions) improvements in service outcomes occurred.

In this article, we set out to test if complexity leadership leads to higher efficiency. Considering what has been described so far, we argue that the enabling leadership function will be essential for the emergence of an efficient leadership system. We hypothesize that the more efficient leadership systems will be those where the enabling leadership function is explicitly encouraged. To test this hypothesis, we conducted a 2-hour controlled simulation study, with groups of five individuals, running city simulations in SimCity4.

## Materials and Methods

### Participants

Participants in this study were graduate students from the Universidade de Lisboa, Portugal. Students attending the Master’s in Organizational Psychology (Faculty of Psychology) were invited to participate in this study via a class announcement, and were asked to invite colleagues from other courses, from the Universidade de Lisboa, to enroll. Participants were compensated for the time they volunteered to the simulation. Respectively, Organizational Psychology Master Students were offered an extra credit for their participation and, all other participants received a 10 euro gift-voucher. Although this compensation was not contingent on task performance (i.e., results), it was contingent on participants’ completion of the experimental task. Rewarding participants for their participation, under the condition that they would only be compensated if they completed the experiment could be regarded as a reward for participation as well [20].

A total of 200 individuals were assigned to 40 groups of 5 people each. The participants mean age was 23.06 years old (*SD* = 4.09), 64.5% were female, and 24.5% had had administrative leadership experience. Moreover, 73% of the participants were full time students (40% of which from Psychology) and 27% were also professional workers. All procedures were approved by the Ethics Committee of Faculdade de Psicologia da Universidade de Lisboa, and the simulations were conducted according to their ethical guidelines.

### Procedure

**Fig 1:**
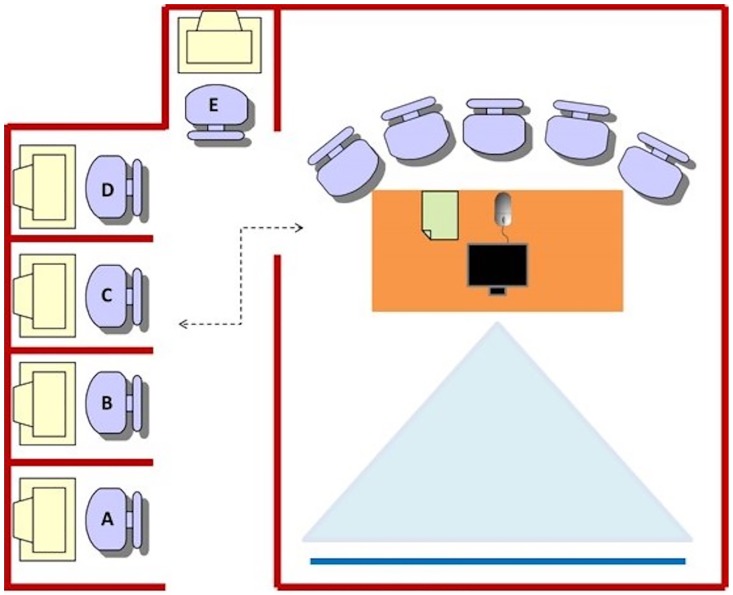
Experiment room.

First of all, it should be noted that only one group at a time participated in the simulation. This means that each participating group had the simulation room (Fig 1) for themselves during the simulation, and that a total of 40 simulation sessions were held to collect all data. Hereupon, when participants first arrived, they were greeted and directed to a group table where they filled in the informed consent and received a letter identification tag.

To give the participants the basic skills to perform the team’s simulation tasks successfully, they were first given 30 minutes to perform four tutorials on single computers (“Get Started”, “Making money”, “Big City” and “Rush Hour”). After participants completed the training assignments, they returned to the group table to begin the team part of the simulation. During the team simulation task, the game was displayed using a projector directed towards a white wall, and the chairs around the table were displayed in a manner that allowed the five participants to see the game and each other. To prevent the person with more experience, or the one with more dominance oriented traits, from being in charge of the computer mouse and always controlling the execution of all the decisions, participants were randomly assigned the control of the computer mouse as well as the keyboard for each team task they had to perform. The simulation included 4 team tasks. Before the beginning of the simulation, the experimenter recommended participants to work as teams and to share knowledge in the decision making process in order to increase population growth by managing and improving the cities funds, considering: the amount of money per capita, the air pollution index, the education level, and life expectancy. To each task, we randomly assigned one of four cities:

- City 1 (Konradshohe Knut). Medium size map that includes, on its left side (from top to bottom), an undeveloped zone, an industrial area with different production facilities, and a residential area with housing, schools and other facilities. On its right side, it includes an undeveloped region, separated from the main city region by a river. Regarding its financial status, City 1 starts with §834 Simoleons, the SimCity currency. It has the largest population (15,025 people) of all of the cities used in the simulation. The city’s main problems concern the environment and the land value.
- City 2 (Tagel Madeline). Small size map that includes, on its left side, mostly farms and other agricultural infrastructures, and in the center, a small residential area. At its bottom, there is a small area of water, and the right side of the map corresponds to an undeveloped area. This city starts with §12,223 and a small population (2,574 people). The city’s main problems are related to a lack of water supply, waste disposal and proper roads.
- City 3 (City Spandau Hans). Large size map that includes, in its bottom left area, an industrial zone, and in its top right area, a residential zone. The top left and the middle part of the map are predominantly undeveloped areas, and the right part of the map is characterized by a water area with an undeveloped zone in the middle. This city starts with §69,986 and no population. The city’s main problems concern the environment and are related to the lack of energy and, low health, education and, safety levels.
- City 4 (City Kensington Beto). Large size map with no water areas and an undeveloped region in the upper part of the map. It is composed of two residential areas, one in the middle left and one in the bottom center of the map. Additionally, it also includes an industrial area in the top center part of the map. This city starts with §31,750 and a medium size population (7,633 people). The city’s main problems regard the lack of energy, the environment, low levels of safety and education, and diminished land value.

Regardless of the task, the difficulty level of each city remained the same (3 stars).

Task 1 was defined as a baseline task where no instructions regarding the leadership system were given. Task 1 was kept constant for all participating groups, and was operationalized as Leadership System 1 (LS1). It also aimed to recreate organizational systems where leadership rules are not clearly defined. In the case of tasks 2, 3 and 4, participants were given a brief summary report that described the leadership behaviors that were known to be more efficient in the management of cities such as the ones they were about to be assigned to. The attribution of the reports worked as initial conditions for the emergence of different leadership system arrangements based on complexity leadership theory. For each leadership condition, respectively from tasks 2 to 4, two leadership functions were combined. The combination was established considering the conceptual work of Uhl-Bien and Marion [5] and their propositions regarding how enabling leadership promotes the entanglement between the administrative and the adaptive functions. Participants were presented with one of three sheets informing them that the city that they were about to manage was better managed when the individuals who were part of the city’s management team displayed paired combinations of complexity leadership’s functions behaviors. In tasks 2, 3 and 4, participants were informed that “Cities like the one that you will manage have been better managed when the teams that run them have individuals who perform the following functions.” Participants then read a paired combination of the description of each function, without being told the theoretical name of the functions that they were reading: leadership system 2 (LS2, Adaptive—Enabler), leadership system 3 (LS3, Administrative—Enabler), and leadership system 4 (LS4, Administrative—Adaptive). For instance, instead of administrative function, they would read “function 1” followed by its behavioral description. Please recall that the coding LS1 was used to describe task 1, which served as a baseline task where no instructions were given regarding leadership. The description of each leadership function, read by each participant, was as follows:

1. Administrative function: Individuals who are formally responsible for managing their own work unit (i.e., team, department, organization). These individuals are responsible for defining how their co-workers organize themselves to carry out their tasks. Moreover, it is their responsibility to structure their co-workers’ activities. Individuals with an administrative function identify ways to perform the duties as required and lead the decision-making process. Additionally, they control the performance and results of those who depend on them. To accomplish this, they have to give feedback to employees, motivate them, and inspire them, hence creating a shared vision of what is expected of each person.
2. Enabling function: Individuals who do not have a formal management or control role in their work unit (i.e., team, department, organization). However, in their work unit, these individuals distinguish themselves by being able to ease the communication between all people by stimulating and arguing, in alignment with the objectives and vision of the organization. They do this in favor of the implementation of ideas that potentially generate innovative/ adaptive responses (e.g., new products, services, solutions). These colleagues help their colleagues to turn ideas into solutions.
3. Adaptive function: Individuals who do not have a formal management or control role in their work unit (i.e., team, department, organization). These individuals distinguish themselves by informally realizing the adaptive demands that their work unit faces or may face. In addition, they intentionally engage in learning activities that allow them to change their troubleshooting, in this way aiding in the promotion of proactive or reactive innovative solutions that enable their work unit to adapt to unexpected or adverse situations.

In short, in task 1, all participating groups were assigned to the LS1 condition, in tasks 2, 3, and 4 we randomly assigned participants one of the three paired combinations of complexity leadership function behaviors (LS2, LS3, and LS4). Furthermore, throughout tasks 1 to 4, we randomized the order by which the cities were assigned to participants. Each task lasted 12 minutes, with time being displayed on a PC screen using the website Online StopWatch (http://www.online-stopwatch.com/fullscreen-stopwatch/). These 12 minutes corresponded to a time lag of 2 years in the game. After the fourth and final simulation (task 4), the participants were excused from the room. After completing the study, participants were debriefed and received instructions to avoid sharing any information regarding their participation in the study with other colleagues.

### Tasks

Teams were tasked with managing a set of four pre-existing cities in SimCity 4. Because SimCity 4 default options only allow for one single player, teams were given the role of City Mayor and were told that they were collectively responsible for making and implementing decisions regarding all aspects of the different cities’ management. All teams were given the goal of increasing population growth by managing and improving the cities funds, considering: the amount of money per capita, the air pollution index, the education level, and life expectancy. To accomplish this, they could carry out a number of management decisions, for example: set tax rates, construct buildings and power grids, or improve public transportation. Although teams were free to choose and act as they saw fit, only one participant was allowed to use the mouse and keyboard to implement the decisions made by the team. SimCity 4 allows for three different game modes (God, Mayor and MySim) and speeds (Turtle, Rhino and Cheetah). In this study participants played in “Mayor Mode” and “Rhino Speed”. This decision was based on a pre-test that concluded that this mode and speed were the most adequate to allow the participants to achieve the goals set for the task considering the time restriction imposed.

### Materials

*SimCity 4*. We utilized the pc-game SimCity4 Deluxe Edition (EA Games, 2004, which previous research has shown that it can be used successfully with regard to group tasks [21]. SimCity4 is a city-building game in which users build, design and govern a metropolitan city with all of the amenities available in real-life cities and where all changes ultimately affect the desirability of the city and the funds available. Organizational systems are exposed to external coordinating constraints and demands. Complexity theory suggests that feedback as a form of coordination constraint is necessary for the emergence and survival of CAS. Feedback facilitates efficient coordination amongst agents because feedback informs them of how well the system is being managed [12]. In this study, feedback is important because it allows for cost control, population growth, focusing efforts, allocating resources, and planning. In fact, SimCity4 allows the agents to be exposed to continuous feedback regarding their own performance during the task, but not information about the other participating teams’ performance. In this study, the creation of external conditions for the emergence of CAS was made possible by the use of SimCity4. *Complexity leadership functions identification items*. After participants had completed each collaborative task they were asked to match each other team members’ behavior with one of the three complexity leadership functions. These functions were presented by means of three simple sentences summarizing the main role that each participant had during the task: administrative leader (“Person with recognized formal authority”), adaptive leader (“Person with a problem solving role”), enabling leader (“Person that enables relations”), and not-applicable (“None of the above mentioned”). This procedure allowed us to identify the proportions of individuals displaying each complexity leadership function within teams. Each member was labeled with the function that the majority of the team attributed to him in the task described above.

## Results

**Fig 2:**
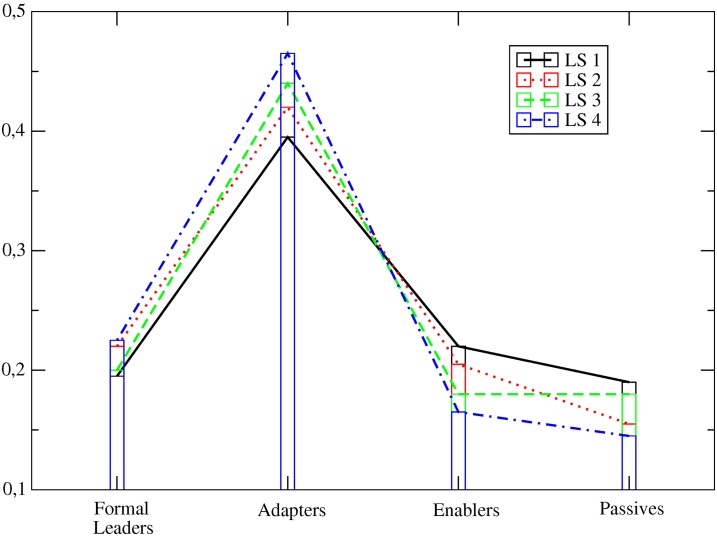
Constellations of complexity leadership functions. Constellations of complexity leadership functions that were found for each leadership system (LS), obtained from the distribution of roles as evaluated by all participants within a group to which a specific LS instruction was given.

In this study we hypothesized that the more efficient organizational systems would be those in which the enabling leadership function is explicitly encouraged. As a first step to test this hypothesis, we examined the CAS compositional structures (i.e., the proportion of individuals with specific complexity leadership functions) that emerged within the team. To accomplish this, we used the evaluation that each team member made regarding group members’ complexity leadership described functions. Fig 2 shows these results. With respect to the comparison of the CAS compositional structures that emerged within each task/trial, we run Qui-square tests to compare the proportion of functions within each team, to see if they were significantly different. We found difference in the proportions between the compositional structures for the enabling and formal functions, but not for the adaptive function. We found that the proportion of enablers was significantly greater for LS2 when compared to LS3 (χ^2^ = 5.1; p = 0.02) and to LS4 (χ^2^ = 3.1; p = 0.07). We also found that the proportion of formal leaders was significantly lower for LS2 when compared to LS3 (χ^2^ = 2.9; p = 0.08) and to LS4 (χ^2^ = 3.9; p = 0.04). These findings, although not binding, support the idea that the enabling function may play a special role in the system.

For the LS1 situation, the number of individuals with enabling and “non-applicable” (individuals to whom none of the leadership function descriptions were related) roles was higher than in LS2, LS3 and LS4; whereas the number of individuals with formal leadership (i.e., administrative function) and adaptive roles was the lowest. The LS2 situation promoted the second highest occurrence of administrative and enabling functions. The LS3 situation clearly promotes a higher frequency of individuals with adaptive and passive functions. Finally, LS4 enhances administrative and adaptive functions. We take these constellations as the correct ones and assume that the manipulations made in the beginning of each task triggered the formation of such constellations. The next step was to establish a quantitative approach for ascertaining which of these constellations is more efficient. In the laboratorial task, the goal was to test how different complexity leadership systems (*i*.*e*. LS2 to LS4) contribute to improve cities’ overall development.

In SimCity, the city development is characterized by variables such as (*i*) the amount of money per capita (*i*.*e*. the difference between actives and passives divided by the total number of citizens), (*ii*) the air pollution index, (*iii*) the education level, and (*iv*) life expectancy. For each variable we collected one hundred data points. A first approach for collecting all of these variables in a quantity that properly measures the efficiency would be to simply sum all of them because they are all positive quantities. Large values of such a sum would indicate large development strength, whereas small values would be interpreted as time instants with weaker development strength. Such an approach, however, is correct only under the assumption that all of the variables are uncorrelated, an assumption that in our case is not realistic. To properly define what we call the development strength of one given team at a particular time during the simulation experiment, we must take all four variables, *X*_i_(*t*), at that particular time *t*, together with the correlation (Pearson) coefficient *ξ*_*ij*_ between each pair of variables, *X*_*i*_ and *X*_*j*_. The coefficient is defined as

$$\xi_{ij}=\frac{\frac{1}{L}\sum_{t=1}^{L}\left(X_{i}\left(t\right)-{\bar{X}}_{i}\right)\left(X_{j}\left(t\right)-{\bar{X}}_{j}\right)}{\sigma_{I}\sigma_{J}}$$ (1)

with L the total number of instants tracked during the simulation experiment and ${\bar{X}}_{i}$ and σ_*i*_ are the mean and the standard deviation of variable *X*_*i*_ respectively:

$${\bar{X}}_{i}=\frac{1}{L}\Sigma_{t=1}^{L}X_{i}\left(t\right),$$ (2a)

$$\sigma_{i}=\sqrt{\frac{1}{L-1}}\Sigma_{t=1}^{L}{\left(X_{i}\left(t\right)-{\bar{X}}_{i}\right)}^{2}.$$ (2b)

A proper development strength measure *F*(*t*) shall sum cumulatively the contribution of each pair of variables in a way that, when *ξ*_*ij*_ = 1, only one property, either *X*_*i*_ or *X*_*j*_, is taken, whereas when *ξ*_*ij*_ = 0 the entire sum *X*_*i*_ + *X*_*j*_ is taken. For all other intermediate cases a sum weighted by the correlation strength is considered. All of these requisites lead naturally to a development strength defined as

$$F\left(t\right)=\frac{2}{N\left(N-1\right)}\Sigma_{i,j=1}^{N}\frac{X_{i}\left(t\right)+\left(1-\left|\xi_{ij}\right|\right)X_{j}\left(t\right)}{2-\left|\xi_{ij}\right|}.$$ (3)

**Fig 3:**
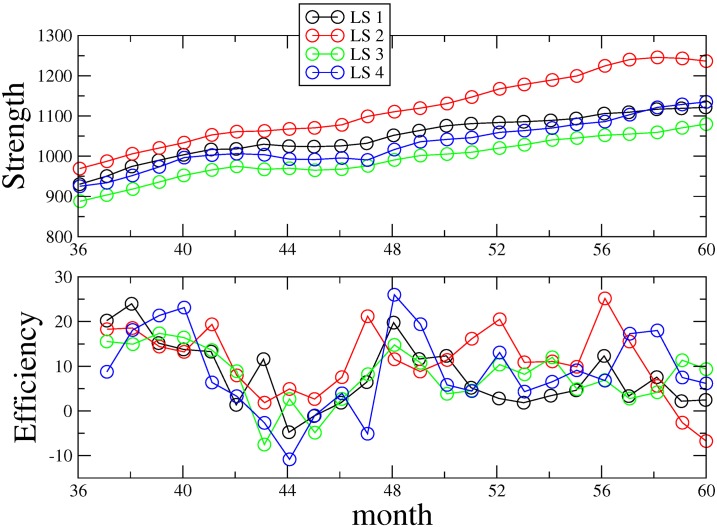
Strength and efficiency of leadership systems across time. Analysis of the complexity leadership functions for each LS through a measure of (a) its strength *F*(*t*) (see Eq 3) and of (b) its efficiency *E*(*t*) (see Eq 4). Time is sampled in units of one month.

In Fig 3 it is shown that when LS are presented, there is a clear tendency to increase development strength. Regardless of the LS presented to each team at the beginning of each task, an increase was observed in time corresponding to an increase of the quantity *F*(*t*). Furthermore, it is also important to determine the way each team manages the city’s construction development rate considering increase and acceleration. Such behavior can be demonstrated by the time derivative of the development strength, namely:

$$E\left(t\right)=\frac{dF}{dt}∼\frac{\Delta F}{\Delta t}=\frac{1}{\Delta t}\left(F\left(t+\Delta t\right)-F\left(t\right)\right).$$ (4)

Here a note on the possibility of learning effects is necessary. While the learning effect is not possible to separate from our results we may argue the following. Since all participating groups were randomly assigned to the tasks (except task 1 which all groups completed first) and the order by which the cities were presented was also randomized, one should expect that the presence of a learning effect in our simulation experiment would reflect equal results for all conditions. Since we observe a non-negligible difference between the four strategies, we can therefore claim that the difference may be even stronger if one removes the learning effect from our analysis.

A team can start with an already large development strength but will not be able to increase further this strength. In such cases, their efficiency *E*(*t*) is, according to (Eq 4), exactly zero. On the contrary, a team that starts with a city that is poorly developed but that is able to increase significantly the development of that city will see a high efficiency *E*(*t*). In our calculations, we use always Δt = 1 month. Although in Fig 3a it is clear that LS2 leads the largest development strength, it is not immediately clear from Fig 3b which LS corresponds to the best efficiency. To properly uncover the most efficient strategy, we computed, for each LS, the efficiency mean *<E>* and standard deviation σ_*E*_ and evaluated the mean-standard deviation ratio.

**Fig 4:**
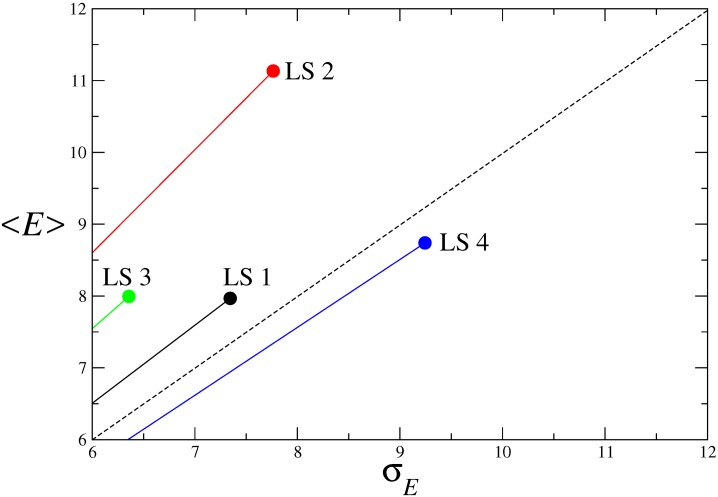
The efficiency of leadership systems. Efficiency of leadership systems, by evaluating the mean-variance of the efficiency *E*(*t*) (see Eq 4). Points above the dashed diagonal, marking <*E*> = σ_*E*_, are the ones having a statistical significant efficiency. From those one clearly sees that the one evidencing higher mean-variance quotient is LS 2, which therefore can be taken as the best strategy for maximum efficiency.

This approach is usually called the mean-variance approach and aims to show two things. First, which rules are indeed efficient? Second, among these rules, which is the most efficient? To answer these questions, we take the efficiency mean as the typical efficiency value for a given LS and the standard deviation associated with the statistical error. Efficient LS are those with a mean efficiency larger than the associated standard deviation (*<E>* > σ_*E*_) because only those show a non-zero efficiency within statistical fluctuation. Fig 4 indicates that only LS4—the one emphasizing the role of adapters and formal leaders—is shown to be inefficient (*i*.*e*. with a negative efficiency within statistical error).

Regarding all of the remaining rules, clearly, LS2 is the one that presents the largest mean variance ratio (Fig 4). This result indicates that LS2 is the best rule for maximizing efficiency (LS1 M = 7.97, SE = 7.37, 95%CI 7.51, 8.43; LS2 M = 11.13, SE = 7.76, 95% CI 10.64, 11.62; LS3 M = 7.99, SE = 6.35, 95% CI 7.59, 8.39; LS4 M = 8.96, SE = 7.84, 95% CI 8.71, 9.21).

Concerning the assumptions behind the mean-variance approach a note is necessary at this point. As one knows the mean-variance approach was introduced in the context of finance in 1952 by Harry Markowitz [22] and it is known as Modern Portfolio Theory. Modern Portfolio Theory is based in the mean-variance analysis under the following assumptions:

1. investment returns are normally distributed, guaranteeing that only returns, variances and covariances are needed to derive the optimal portfolio;
2. investors prefer higher return for a given level of risk, accounted by the respective variance;
3. expected returns, variances and covariances of all assets are known by all investors; and
4. in each transaction there are no costs of any kind.

Therefore, such approach is only possible under the assumption that the statistical variable, in our case the efficiency—is normally distributed. There are however situations where non-Gaussian behavior is handled in a similar way, such as in turbulent flow which are often characterized by the so-called turbulence intensity [23] (ratio between the velocity field fluctuations, i.e. standard deviation, and the respective mean velocity) or in global finance, which sometimes handles extreme scenarios such as financial global crises or sudden policy changes in the system. Extreme events would imply non-Gaussian features, such as fat tails and asymmetry around the first central moments. In the context of organizations and leadership behavior one should also take into account the limitation of such an approach when dealing with more abnormal working situations. For such scenarios other specific group strategies may be the optimal ones, a question that is outside the scope of our work. In our work no extreme scenarios were observed.

To conclude our analysis and test our research hypothesizes we finally tested whether groups significantly differed in terms of efficiency. Firstly, we performed a one-way ANOVA test. The results show that overall group efficiency significantly differed across leadership systems (*F* (3, 3836) = 35.467, p = .00). Secondly, we performed a series of *t*-tests to compare which leadership systems significantly differed regarding efficiency. The results in Table 1 show that leadership system 1 and leadership system 3 did not differ from each other, whereas all other leadership systems significantly differed in terms of their level of efficiency. These results are in line with complexity leadership theory [5] and offer support to our initial hypothesis: Leadership systems in which enabling leadership behaviors are formally encouraged are more efficient than those in which no particular instructions are given, or in which enabling leadership behaviors are not formally acknowledged as being relevant.

**Table 1:** T-tests for leadership systems’ efficiency.

	score differences	t	*d*.*f*.	*p*
**LS1-LS2**	-3.16	-9.166	1911.6	0.00
**LS1-LS3**	-0.02	-0.064	1878.6	0.474
**LS1-LS4**	-0.77	-2.022	1824.1	0.021
**LS2-LS3**	3.14	9.703	1845.2	0.00
**LS2-LS4**	2.39	6.137	1861.9	0.00
**LS3-LS4**	-0.75	-2.073	1699.1	0.019

## Discussion

In this study, we examined leadership under complexity theory. Our findings show that a highest work group efficiency can be achieved by those groups where complexity leadership theory is applied. To the best of our knowledge, this study makes a first formal and deliberate attempt to empirically examine complexity leadership theory. Our research findings suggest that for groups behaving like complex adaptive social systems, more efficient outcomes can be achieved when the administrative, adaptive, and enabling functions are present in commensurate levels. Additionally, the results show that higher efficiency is achieved in groups where the number of individuals with enabling function is moderate, compared to low efficiency groups that display extremely high and low numbers of individuals with enabling functions. Our results also suggest that higher efficiency can be achieved in systems where enabling leadership and adaptive leadership functions are simultaneously encouraged. In contrast, in systems where enabling leadership and administrative leadership functions are simultaneously encouraged efficiency is lower. Moreover, our results also suggest that under conditions where the enabling leadership function is not formally encouraged teams perform poorly. Going back to the story of the Katrina in 2005, it was the enabling and adaptive behaviors of those living in the area that promoted the emergence of a CAS that offered an almost immediate solution to the situation. The CAS resulted in a communication and rescue network led by Cajun boat men, that coordinated with other citizens that improvised temporary hospital facilities to shelter those that were injured, lost, or homeless [2, 3].

According to Uhl-Bien and Marion [5], when enabling behaviors are encouraged within the leadership system, even when agents are adaptive or administrative oriented, the simple fact that enabling leadership behaviors are stimulated may facilitate the use of enabling leadership behaviors by these agents. When adaptive and administrative functions are effectively entangled, the role of enabling leadership becomes hard to distinguish from the other two functions. In other words, adaptive and administrative leaders can also be enabling leaders. However, in the administrative and adaptive behaviors simulation, we recreated a more bureaucratic organizational structure where formal leaders give orders and adapters implement them. In line with complexity leadership theory [7], when no enabling action is encouraged, but only administrative and adaptive behaviors are encouraged, the leadership systems might have more difficulty in guaranteeing the ease of communication that is promoted by the enabling behaviors. We found that the best performing leadership configuration, LS2, had significantly lower proportion of formal leaders than LS4 and LS3 configurations. This may suggest that in this kind of complex adaptive systems, formal leadership functions seem to be necessary for performance but in a moderate amount. This might explain why efficiency was lowest for LS4 leadership systems. In addition, we also found that LS2 had significantly more enablers than LS3 and LS4. A complementary explanation could be that the enabling function facilitates learning in the system because it facilitates the transformation of ideas generated by adapters into effective solutions implemented by formal leaders. Such effect, to be true, could help us understand why the learning curve is steeper for LS2 than for the other leadership systems (Fig 3a).

Our results also indicate that there were individuals whose team members did not identify them as engaging in any of the simulation tasks functions assigned to the team. They did not attribute any of the complexity leadership behaviors being formally encouraged before each task. This result suggests that other functions might exist in the system, as it might be the case of a passive function [13]. Organizational literature identifies it as social loafing, which is the phenomenon that characterizes people who exert less effort to achieve a goal when they work in a group than when they work alone [24]. Social loafers are often perceived as passives in the sense that other group members regard them as not proactively contributing to the group purpose. In another vein, this passive leadership behavior has been found in another type of evolutionary games where the two types of leadership emerged—active or passive—depending on the environment characteristics, the group members’ personalities and a favorable initial random position within the environment [25].

Although our results might seem to suggest that an optimal proportion of complexity leadership functions exist, this is not the case. By defining leadership performance rules at the beginning of each new task, we allowed complexity leadership functions to develop as CAS. Our results only tell that in leadership systems where enabling leadership is formally encouraged, the system will achieve higher efficiency than in systems where nothing is said, or enabling leadership is not encouraged. In line with complexity leadership theory, efficiency can only be achieved if managers enable, rather than control, informal network dynamics (i.e., enabling and adaptive functions). These research findings bring further support to the growing evidence that efficient leadership emerges as CAS, and how leadership is thought and practiced should not be restricted to individual-centric approaches [24].

Given that our study was conducted in the laboratory, and that participants were organized in small groups (even though a large amount of nowadays so called micro companies are composed by no more than 5 individuals), the generalizing of our findings to large scale organizational systems should be made with caution. Finally, we believe that the outcomes of this study are also of interest for the practice of leadership. Learning that the encouragement of enabling leadership behaviors within groups and organizational leadership systems promotes higher efficiency is a valuable insight to inform managers and employees about what leadership strategies they ought to implement in case they wish to achieve maximum efficiency. Although humans tend to organize hierarchically when they are ought to perform complex tasks that cannot be attained by single individuals (e.g., creating a company), and hierarchical forms of organizing are traditionally associated with centralized control [26], it might be that educating leaders and employees for complexity leadership will help hierarchical organizational structures to become more resilient to change.

## Supporting Information

S1 File GroupsGoalOutcomes_DataSet. (ZIP)

S2 File LeadershipFunctions_DataSet. (ZIP)
